# Single Cell Mass Cytometry of Non-Small Cell Lung Cancer Cells Reveals Complexity of In Vivo and Three-Dimensional Models over the Petri-Dish

**DOI:** 10.3390/cells8091093

**Published:** 2019-09-16

**Authors:** Róbert Alföldi, József Á. Balog, Nóra Faragó, Miklós Halmai, Edit Kotogány, Patrícia Neuperger, Lajos I. Nagy, Liliána Z. Fehér, Gábor J. Szebeni, László G. Puskás

**Affiliations:** 1 Avicor Ltd., H6726 Szeged, Hungary; r.alfoldi@astridbio.com; 2 University of Szeged, PhD School in Biology, H6726 Szeged, Hungary; balog.jozsef@brc.mta.hu; 3 AstridBio Technologies Ltd., H6726 Szeged, Hungary; 4 Laboratory of Functional Genomics, HAS BRC, H6726 Szeged, Hungary; n.farago@avidinbiotech.com (N.F.); halmaim@yahoo.com (M.H.); kotogany.edit@brc.mta.hu (E.K.);; 5 Avidin Ltd., H6726 Szeged, Hungary; l.nagy@avidinbiotech.com (L.I.N.); l.feher@avidinbiotech.com (L.Z.F.); 6 Research Group for Cortical Microcircuits of the Hungarian Academy of Sciences, Department of Physiology, Anatomy and Neuroscience, University of Szeged, H6726 Szeged, Hungary; 7 Department of Physiology, Anatomy and Neuroscience, Faculty of Science and Informatics, University of Szeged, H6726 Szeged, Hungary

**Keywords:** single cell mass cytometry, single cell proteomics, non-small cell lung cancer, three-dimensional tissue culture

## Abstract

Single cell genomics and proteomics with the combination of innovative three-dimensional (3D) cell culture techniques can open new avenues toward the understanding of intra-tumor heterogeneity. Here, we characterize lung cancer markers using single cell mass cytometry to compare different in vitro cell culturing methods: two-dimensional (2D), carrier-free, or bead-based 3D culturing with in vivo xenografts. Proliferation, viability, and cell cycle phase distribution has been investigated. Gene expression analysis enabled the selection of markers that were overexpressed: *TMEM45A, SLC16A3, CD66, SLC2A1, CA9, CD24,* or repressed: *EGFR* either in vivo or in long-term 3D cultures. Additionally, TRA-1-60, pan-keratins, CD326, Galectin-3, and CD274, markers with known clinical significance have been investigated at single cell resolution. The described twelve markers convincingly highlighted a unique pattern reflecting intra-tumor heterogeneity of 3D samples and in vivo A549 lung cancer cells. In 3D systems CA9, CD24, and EGFR showed higher expression than in vivo. Multidimensional single cell proteome profiling revealed that 3D cultures represent a transition from 2D to in vivo conditions by intermediate marker expression of TRA-1-60, TMEM45A, pan-keratin, CD326, MCT4, Gal-3, CD66, GLUT1, and CD274. Therefore, 3D cultures of NSCLC cells bearing more putative cancer targets should be used in drug screening as the preferred technique rather than the Petri-dish.

## 1. Introduction

Development of single-cell analytical techniques extends our understanding of cell population heterogeneity and enables the identification and characterization of highly specialized rare cell types [1]. Single cell genomics (e.g., single cell RNAseq) and single cell proteomics (e.g., mass cytometry) have revolutionized our knowledge about the co-ordination of different cell types in tissue microenvironments unveiling their characteristic protein patterns [2,3]. Although image-based single cell analysis has also been developed [4], single cell mass cytometry has been adopted to investigate millions of cells per sample and it offers multi-dimensional data analysis with the characterization of multiple proteins at single cell resolution [5].

Lung cancer accounts for the majority, 25% of all cancer-related deaths worldwide and the 5-years overall survival at 17.7% has achieved very little progress in the last decades [6]. Adenocarcinomas account for the majority, 40% of all lung cancer histological types [7]. Here, we focus on the mass cytometric single cell analysis of a non-small cell lung carcinoma (NSCLC) model, the A549 adenocarcinoma cells. The heterogeneity of immune subsets infiltrating non-small cell lung cancer has been previously published based on mass cytometric profiling [8]. Here, we focus on marker expression of lung cancer cells obtained from different culture conditions at single cell resolution.

Organoid cell culturing revolutionized cell biology since in vitro three-dimensional (3D) multicellular spheroid models mimic better the physiology of complex tissues compared to conventional two-dimensional (2D) monolayer cultures [9]. For a more successful treatment of lung cancer, a better understanding of cancer development in the tissue microenvironment and further improvements in in vitro experimental techniques are needed [10]. Ideal models systems, for screening of novel drug candidates should mimic the molecular, functional and histopathological complexity of in vivo tumors more accurately. Although multi-cellular tumor spheroid models were introduced in the early 1970s [11,12], their implementation in the pre-clinical phase of drug development was neglected resulting in numerous failed clinical trials [13].

Currently, there is a wide variety of techniques for three-dimensional cell culture methods: specially designed incubators, tubes, microcarriers and growing matrices [13]. Various extracellular matrix (ECM) components and their homologues (collagen, gelatin, etc.) are used to facilitate the adhesion of cells to the carriers [14,15]. Investigation of tumor spheroids revealed, that the core of the spheroid was similar to in vivo conditions. Three-dimensional spheroids mimic the organoid of a solid tumor, while 2D culture methods fail to represent different tissue areas within tumors, such as proliferating, quiescent and necrotic core zones [16]. So far, the traditional in vitro screening of drug candidates in cell-based assays has been based on adherent cultures (2D assays) [17,18], but several studies reported the application of cancer multicellular spheroids for screening and target identification [14,19,20,21,22]. It has been shown by us [23,24] and others [25,26] that the cells grown on 2D surfaces demonstrate higher drug sensitivity. The monolayer culture of the human breast cancer cell line MCF-7 showed 2.6-fold higher accumulation of doxorubicin, paclitaxel, and tamoxifen compared to three-dimensional cultivation on porous biodegradable polymeric microparticles [27]. Only 26% of cells in 3D reached the same concentration of drugs as the cells treated in 2D dishes because the synthesis of ECM components was more intense in 3D cultures [27]. Furthermore, cells in monolayer culture not only have higher sensitivity to anticancer drugs upon treatment but they become quiescent and decrease proliferation rate and increase apoptosis more quickly than 3D models or in vivo tumors [10,13].

In this study, we characterized 2D, 3D and in vivo models by a state-of-the-art single cell mass cytometry technique. Here, we show that A549 lung cancer cells grown by organic scaffolded or scaffold-free 3D culturing methods represent the gene and protein expression patterns of in vivo tumors better than standard 2D cultures.

## 2. Materials and Methods

### 2.1. Two-Dimensional (2D) Cell Culture

The NSCLC cell line A549 was purchased from the ATCC collection. Since cells were maintained in our laboratory, cell identification of A549 cells was performed by Microsynth AG (Balgach, Switzerland). Cells were maintained in DMEM/F12 (DMEM, PAN^TM^ Biotech; F12 Nut mix, Gibco, Thermo Fisher Scientific, Waltham, MA, USA) containing 4.5 g/L glucose, 10% fetal bovine serum (FBS, Gibco), 1X GlutaMAX (Gibco) 1% PenStrep antibiotics (Penicillin G sodium salt, and Streptomycin sulfate salt, Sigma-Aldrich, St. Louis, MI, USA). The cells were cultured in standard tissue culture Petri-dish (2D monolayer, 10 mm diameter dish, Corning Life Sciences, Corning, NY, USA); T-75 flasks (2D TC, tissue culture flasks T75 flask, TPP, Trasadingen, Switzerland) or collagen type I covered T-75 flasks (2D Coll, tissue culture flasks T75 flask, TPP) for time points day 4 and 9 at maximum 80% confluence at standard atmosphere of 95% air and 5% CO_2_ (Sanyo, Osaka, Japan). At about 80% confluence cells were washed, harvested with trypsin (Sigma-Aldrich) and seeded into new dish.

### 2.2. T-75 Flask Surface Coating

The collagen type I covered T-75 (2D Coll, tissue culture flasks T75 flask, TPP) flasks were coated by 4 mL of 0.1% collagen (Collagen Type I, Sigma-Aldrich) diluted in sterile phosphate-buffered saline (PBS, Sigma-Aldrich) at room temperature. After 30 min, the flasks were washed with sterile water and dried under sterile hood before use.

### 2.3. Three-Dimensional Microcarrier Coating

Two types of microcarriers were used for 3D culturing. The Cytodex 3 (Sigma-Aldrich, 3D Cytodex3) dextran microcarriers were purchased as prefabricated and precoated with denatured porcine-skin collagen. The other type of microcarrier, Nutrisphere (Hamilton, Reno, NV, USA) was coated by collagen (collagen type I, Sigma-Aldrich) in our laboratory (3D Nutrisphere). 2 mL of Nutrisphere magnetic microcarriers were transferred into silanized vials (Sigma-Aldrich) and washed with sterile water and MOPS buffer solution (0.1M MOPS, pH 5.0, Sigma-Aldrich). The activation of microcarriers was performed with EDC/NHS reagent: 600 mM EDC (1-Ethyl-3-(3-dimethylaminopropyl) carbodiimide hydrochloride, Sigma-Aldrich) and 200 mM NHS (N-hydroxysulfosuccinimide, Sigma-Aldrich) dissolved in 0.1 M MOPS buffer, pH 5.0 (Sigma-Aldrich). After 20 min surface activation at room temperature the microcarriers were washed with phosphate-buffered saline at neutral pH. The coating was carried out on a shaker with slowly agitation in 1 mL of 5 mg/mL collagen type I solution and incubated overnight under shaking condition at room temperature. The next day, the supernatant was discarded, and the collagen-coated microbeads were washed twice and finally resuspended in PBS, sterilized by autoclave (121 °C for 15 min) and stored at 4 °C. Cells were grown on microcarriers at equivalent surface/volume ratio to T75 flasks.

### 2.4. Three-Dimensional (3D) Cell Culturing Using Bench-top Incubator System

A549 cells (3D Cytodex3, 3D Nutrisphere, and carrier-free spheroids) were cultured in specially designed LeviTubes in the bench top bioreactor-incubator hybrid (BioLevitator^TM^, Hamilton). Cells were grown on microcarriers at equivalent surface/volume ratio to T75 flask. Cytodex3 beads are 175 µm in diameter and surface varies between 200–230 cm^2^/mL. Nutrisphere beads are 65 µm in diameter and surface is around 125 cm^2^/mL. To keep surface/volume ratio equivalent with the 75 cm^2^ of flask, 350 µL Cytodex3 and 602 µL Nutrisphere were used for inoculation, separately. Both for microcarriers and carrier-free spheroids 1 × 10^6^ cells were inoculated. During the cultivation, cells were grown in suspension culture with or without microcarriers with the following setup: Inoculation period was for 5 h: Rotation Pause: 0 s, Rotation Period: 1 s, Agitation Pause: 20 min, Rotation Speed: 50 rpm, Agitation Period: 5 min. Duration: 5 h. Culture period (protocol) was the following for 4 or 9 days: Rotation Pause 0 s, Rotation Period: 1 s, Rotation Speed: 75 rpm. Duration: ∞. LeviTubes were filled up with 40 mL DMEM/F12 media contained 10% fetal bovine serum, 1X GlutaMAX and 1X PenStrep. Culture medium was changed once after 72 h by removing half of the volume and replaced it with fresh media.

### 2.5. Real Architecture for 3D Tissue (RAFT) Culturing

Three-dimensional real architecture for 3D tissue (RAFT) cultures were prepared following the instructions of the manufacturer (Lonza, Basel, Switzerland) as described previously [15]. Briefly, the components of the kit (Type I rat tail collagen, 10X MEM culture medium, neutralization solution) and cell suspension were gently mixed and aliquoted into the 96-well plate (240 μL mix per well, 4000 cells per well) and placed into the CO_2_ cell culture incubator (37 °C, 5% CO_2_) for 15 min to form the collagen hydrogel. The hydrophilic RAFT absorbers were placed onto the top of hydrogels and left for 15 min to absorb the free fluids from the collagen discoids. Finally, the wells were filled up with 200 µL cell culture media, and the plates were placed in the cell culture incubator under standard culture conditions (5% CO_2,_ 37 °C). RAFT cultures were pooled for experiments at equivalent cell load compared to other cell culture methods.

### 2.6. A549 Xenograft Tumor Model

For gene expression measurements 1 × 10^6^ A549 cells in 100 µL FBS free DMEM/F12 were injected subcutaneously into 8-weeks-old NOD SCID mice (Innovo Ltd., Isaszeg, Hungary). A few weeks later when the xenograft tumors reached the volume of 80–100 mm^3^, the mice were sacrificed, and subcutaneous tumor tissues were excised and processed for gene expression analysis.

For mass cytometry (Helios, Fluidigm, San Francisco, CA, USA) measurements the 8-weeks-old NOD SCID mice (*n* = 6) were injected subcutaneously with 1 × 10^6^ A549 cells in 100 µL FBS free DMEM/F12 and the mice were sacrificed at two different time points, three of them after 30 days, when the tumor reached volume of 80–100 mm^3^ (early stage, non-necrotic small tumors) and another three mice after 60 days, when the tumor reached volume of 1000–1200 mm^3^ (late stage, necrotic tumors).

All mouse studies were done in accordance with national and international laws and regulations of animal experiments and were reviewed and approved by the Regional Animal Health Authorities, Csongrad County, Hungary, and by the Joint Local Ethics and Animal Welfare Committee of Avidin Ltd. in possession of an ethical clearance XXIX./128/2013.

### 2.7. Imaging

Digital phase contrast images were taken by the HoloMonitor M3 instrument using phase contrast X10 objective (Phase Holographic Imaging AB, Phiab, Sweden) and the analysis computer (HoloStudio 2.0 software, Phiab, Sweden). Phase contrast images were used as a reference to confirm that the cells were in good condition under the studied period. Changes were analyzed at days 4 and 9.

### 2.8. Cell Proliferation Assay

The proliferation of A549 cells was determined by the fluorescent resazurin (Sigma-Aldrich) assay as described previously [28]. Briefly, an aliquot of all types of cells (6000) pre-cultured in either different 3D or 2D conditions were removed and seeded into 96-well plates (Corning Life Sciences) in DMEM/F12 10 % FBS (Gibco) in order to perform the viability assay every day. Resazurin reagent (Sigma-Aldrich) was dissolved in PBS (pH 7.4) at 0.15 mg/mL concentration, 0.22 µm filtered and aliquoted at −20 °C. We applied resazurin 20 µL stock to 100 µL culture. After 2 h incubation at 37 °C under 5 % CO_2_ (Sanyo) fluorescence (530 nm excitation / 580nm emission) was recorded on a multimode microplate reader (Cytofluor4000, PerSeptive Biosytems, Framingham, MA, USA). Proliferation was calculated with relation to blank wells containing media without cells. (Significance was compared to 2D TC, pairwise. RFU = relative fluorescence unit)

### 2.9. Cell Cycle Analysis

Cells were released from all cultures by digestion with 1 mg/mL collagenase IV (Sigma-Aldrich) for 30 min for 3D and 5 min for 2D at 37 °C, manually shaken in serum-free DMEM (Gibco), 12 RAFT discoids were pooled for one flow cytometric sample. Cell cycle analysis was performed as described previously [29]. Briefly, the cells (50,000) cultured under different conditions were collected, washed with PBS and resuspended in DNA binding buffer (1X PBS, 0.1% tri-sodium-citrate, 10 µg/mL PI, 0.1% Triton X-100, 10 µg/mL RNaseA, Sigma-Aldrich) on days 4 and 9. After 30 min incubation at room temperature cells were acquired on a FACSCalibur cytofluorimeter (Becton Dickinson, Franklin Lakes, NJ, USA), sub-G1 apoptotic population was analyzed on FL3 histograms using CellQuest software (Becton Dickinson). Doublets were gated out for cell cycle analysis which was based on FL2-A/FL2-W dot plots, using Modfit software version 3.2 (Becton Dickinson).

### 2.10. Apoptotic Assay

Cells were released from all cultures by digestion with 1 mg/mL collagenase IV (Sigma-Aldrich) for 30 min for 3D and 5 min for 2D at 37 °C, manually shaken in serum-free DMEM (Gibco), 12 RAFT discoids were pooled for one assay sample. Apoptosis was detected as described previously [30]. Briefly, cells cultured under different conditions were collected and resuspended in Annexin V binding buffer (0.01 M HEPES, 0.14 M NaCl and 2.5 mM CaCl_2_, Sigma-Aldrich) on the 4^th^ and the 9^th^ day. Annexin V-Alexa 488 (Thermo Fisher Scientific, 2.5:100) was added to the cells, which were then kept in dark at room temperature for 15 min. Before the acquisition, propidium iodide (10 μg/mL) (Sigma-Aldrich) was added in Annexin V binding buffer to dilute Annexin V-Alexa 488 5X. Cells were analyzed on a FACSCalibur cytofluorimeter using CellQuest software (Becton Dickinson). The percentage of the FL1 (AnnexinV-Alexa 488, AnnV) negative and FL3 (propidium iodide, PI) negative living cells (AnnV−/PI−), the early apoptotic (AnnV+/PI−), late apoptotic (AnnV+/PI+) and necrotic (AnnV−/PI+) cells were determined.

### 2.11. Profiling of RNAs with High-Throughput, Nanocapillary qRT-PCR

Nanocapillary qRT-PCR was performed as described previously with some modifications [31,32]. The gene expression profile of two-dimensional standard culture of A549 cells (70% confluent in standard tissue culture Petri-dish, 2D monolayer) were compared to xenografts. Supernatant was removed from the 2D monolayer, cells were washed two times by 1 mL of PBS, then 1 mL AccuZol™ Total RNA Extraction Solution was added for homogenization per sample. Solid tumors were removed surgically (at 80 mm^3^ volume), cut into 2 pieces and one half placed into RNAlater (Thermo Fisher Scientific). Tumor tissue was homogenized by Tissue Homogenization Set (Bioneer, Daejeon, Korea). RNA was purified from both 2D and ex vivo samples by Direct-zol^TM^ MiniPrep Plus (Zymo Research, Irvine, CA, USA). The quantity of total RNA was measured by NanoDrop 1000 spectrophotometer (Thermo Fisher Scientific). For nanocapillary qRT-PCR total RNA (2 µg) was converted into cDNA with the High-Capacity cDNA RT Kit (Thermo Fisher Scientific) and without purification the mixture was diluted with RNase-free water. Amplification of the samples was followed in real time with QuantStudio™ 12K Flex System (Thermo Fisher Scientific). To determine the gene expression changes TaqMan^®^ OpenArray^®^ Human Cancer Panel was used. This gene signature panel targets 624 well-defined genes validated as markers for pluripotency, DNA repair, angiogenesis, cell adhesion, apoptosis, and extracellular matrix, as well as genes involved in the cell cycle plus 24 endogenous control genes. The format of the OpenArray^®^ plate allows for 4 replicates to run in parallel per plate (2-2 biological replicates were analyzed on a slide). The cancer panel gene list is available in Supplementary Materials File 1.

The reverse transcribed samples (or water for no template controls) were added to a 384-well plate containing TaqMan^®^ OpenArray^®^ Real-Time PCR Master Mix (Thermo Fisher Scientific) for OpenArray^®^ amplification. The OpenArray^®^ autoloader transfers the cDNA/master mix from the plate to the array through-holes by capillary action. Each subarray was loaded with 5 μL of reaction mix containing 1.2 μL of reverse transcribed cDNA resulting in 33 nL final reaction volume containing 0.8 ng cDNA. The array is manually transferred to the OpenArray^®^ slide case and sealed. The plates were cycled in the OpenArray^®^ cycler under the following conditions: 50 °C for 15 s, 91 °C for 10 min, followed by 50 cycles of 54 °C for 170 s and 92 °C for 45 s.

The QuantStudio 12K Flex software uses a proprietary calling algorithm that estimates the quality of each individual threshold cycle (Ct) value by calculating a Ct confidence value for the amplification reaction. In our assay, Ct values with Ct confidence values below 300 (average Ct confidence of the non-target amplification reactions plus 3 standard deviations) were considered background signals. Higher Ct confidence levels were considered positive and were analyzed further. Normalization was done by using the Ct value of *HPRT1* house-keeping gene (ΔCt = Ct_gene_ − Ct_HPRT1_) and gene expression changes were calculated from two replicates. Data are expressed as ΔΔCt (log_2_) values and were normalized to expression values from cells maintained in standard tissue culture Petri-dish (monolayer); (ΔΔCt (log_2_) = ΔCt_monolayer_ − ΔCt*_in vivo_*). Average values were accepted when the standard deviation (SD) was below 0.5-fold of the average. Expression data (ΔΔCt ± SD, *p*) for 60 selected genes are in Table 2, for 648 genes are available in Supplementary Materials File 1.

### 2.12. Gene Expression Analysis by High-Throughput qRT-PCR

Cells were released from all cultures by digestion with 1 mg/mL collagenase IV (Sigma-Aldrich) for 30 min for 3D and for 5 min for 2D at 37 °C, manually shaken in serum-free DMEM (Gibco), RAFT discoids were pooled for one sample. Reference sample was the standard culture of A549 cells (70% confluent in standard tissue culture Petri-dish, 2D monolayer). High-throughput qRT-PCR was performed as described previously [33]. Briefly, on 4^th^ and 9^th^ day the cells cultured under different conditions were collected, centrifuged (3000  rpm, 5 min) and total RNA was purified using AccuPrep Viral RNA Extraction kit (Bioneer) with a modified protocol. Briefly, cells were lysed with RA1 lysis buffer (Macherey-Nagel, Düren, Germany) and applied to the Viral RNA Extraction binding tube and then washed and eluted with the protocol recommended by the manufacturer. The quantity of total RNA was measured by NanoDrop 1000 spectrophotometer (Thermo Fisher Scientific). Total RNA (6 μg) was converted into cDNA with the High-Capacity cDNA Archive Kit (Applied Biosystems, Foster City, CA, USA) in a total volume of 60 μL.

The qPCR reactions were prepared by the Agilent Bravo Liquid Handling Platform (Agilent Technologies, Santa Clara, CA, USA) according to manufacturer’s recommendations. Each 2 μL reaction mixture contained 6 ng cDNA, 10 pmol gene-specific primers, and 1 μL 2× LightCycler1536 Probes Master (Roche, Basel, Switzerland). List of primers is available in Supplementary Materials File 2. Amplification was performed on the LightCycler 1536 System (Roche) using 64 pre-selected genes. The panel consists of 62 tumor-related genes with two human reference genes (*GAPDH* and *HPRT1*). During the amplification the following protocol was used: 95 °C for 1 min, 60 cycles of 95 °C for 10 s, and 60 °C for 10 s, followed by 40 °C for 10 s final cooling. Data were collected and processed using the LightCycler 1536 SW 1.0 software (Roche). Relative expression of the analyzed genes was normalized to the mean value of the *HPRT1* reference gene (ΔCt = Ct_gene_ − Ct_HPRT1_) and gene expression changes were calculated from four replicates. Data are expressed as ΔΔCt (log_2_) values and were normalized to expression values from cells maintained in standard tissue culture Petri-dish (monolayer); (ΔΔCt (log_2_) = ΔCt_monolayer_ − ΔCt_2D or 3D cultures_). Expression data (ΔΔCt ± SD, *p*) for all 62 genes are available in Supplementary Materials File 3.

### 2.13. Cluster Analysis

Cluster analysis (hierarchic) was performed by Gene Cluster 3.0 software (University of Tokyo, Tokyo, Japan) from expression data (ΔΔCT, log_2_ values) of cells maintained under different culture conditions.

### 2.14. Single Cell Mass Cytometry

A549 cells were maintained under the following tissue culture conditions: long term (9 days) (1) standard tissue culture (10 mm diameter cell culture Petri-dish (Corning, 2D monolayer), (2) grown on Cytodex3 or (3) Nutrisphere beads (3D) and injected into SCID mice to form (4) early and (5) late stage tumors. Cells were washed with PBS and liberated by Accutase (Corning Life Sciences) from 2D monolayer and 3D cultures. Tumor tissues were homogenized mechanically using scissors and tweezers. Small pieces were incubated with Accutase for 60 min at room temperature. Samples were loaded on cell strainer (100 μm in pore size, VWR, Radnoe, PA, USA) and washed by PBS. Cells were counted using Bürker chamber and trypan blue viability dye. Three million cells pooled from three biological replicates were processed for mass cytometry staining in suspension in PBS. Viability of the cells was determined by cisplatin (5 μM 195Pt, Fluidigm) staining for 3 min on ice in 300 μL PBS. Sample was diluted by 1500 μL Maxpar Cell Staining Buffer (MCSB) and centrifuged at 350 g for 5 min. Cells were suspended in 50 μL MCSB and the following antibody mix (Table 1) was added in 50 μL.

The following antibodies were conjugated with metal tags in house: anti-CA9, anti-GLUT1, anti-MCT4 and anti-TMEM45A using Maxpar metal labeling kit strictly according to the instructions of the manufacturer (Fluidigm). Antibodies were titrated prior to the experiment in order to determine the optimal dilution.

Samples after 60 min incubation at 4 °C, antibodies were washed by 2 mL MCSB and centrifuged at 300 g 5 min, two times. The pellet was suspended in the residual volume. Cells were fixed in 1.6 % formaldehyde (freshly diluted from 16% Pierce formaldehyde with PBS, Thermo Fisher Scientific) and incubated for 10 min at room temperature. Cells were centrifuged at 800 *g* for 5 min. Cell ID DNA intercalator (191/193 Iridium, Fluidigm) was added in 1000× dilution in Maxpar Fix and Perm for overnight at 4 °C. Cells for the acquisition were centrifuged at 800 g for 5 min then were washed by 2 mL MCSB and centrifuged at 800 g for 5 min. Cells were suspended in 1 mL PBS (for WB injector) and counted in Bürker-chamber during centrifugation. For the acquisition, the concentration of cells was set to 0.5 × 10^6^/mL in cell acquisition solution (CAS) containing 10% EQ Calibration Beads. Cells were filtered through 30 μm gravity filter (Celltrix, Uppsala, Sweden) and acquired freshly. Mass cytometry data were analyzed in Cytobank (Beckman Coulter, Brea, CA, USA). Single living cells were determined (1 × 10^5^ for 2D and early stage in vivo tumors; 3 × 10^5^ for 3D models and 5 × 10^5^ events for late in vivo). viSNE (visualization of stochastic neighbor embedding) analysis (iterations = 1000, perplexity = 30, theta = 0.5), was carried out on 4.5 × 10^4^ HLA-A,B,C+ events for all samples excluding stromal cells of the A549 xenografts [34].

### 2.15. Statistical Analysis

Statistical analysis was performed using GraphPad Prism 6 (Sandiego, CA, USA) and Microsoft Excel (Redmond, WA, USA). Paired t-test was performed between two groups as indicated in the figure legends. Data were expressed as arithmetic mean ± standard deviation (SD).

## 3. Results

### 3.1. Long-term Growth Curve, Apoptosis, and Cell Cycle Phase Distribution of 3D Cultures

**Figure 1:**
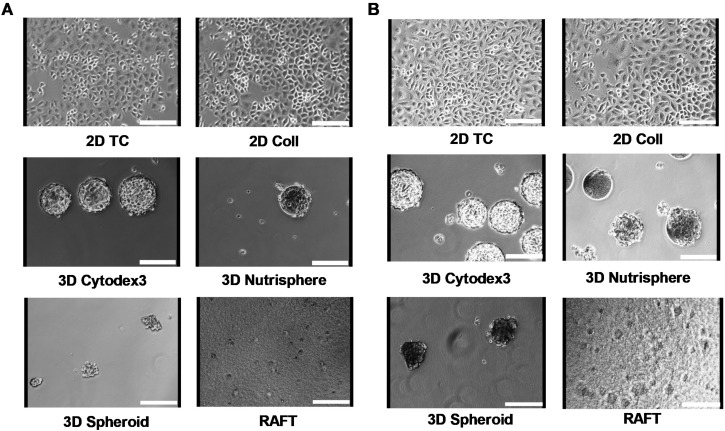
Different culture conditions of A549 human adenocarcinoma cells for 4 days (**A**) and for 9 days (**B**). Cells were seeded at the same surface/volume ratio regarding the different culture conditions as described in Materials and Methods. Images were taken by the HoloMonitor M3 instrument using phase contrast X10 objective. Scale bar: 150 µm.

Non-small cell lung carcinoma A549 cells were cultured under different conditions for 4 days as short term (Figure 1A) and for 9 days as long-term (Figure 1B) in order to analyze whether different incubation times affect growth kinetics, viability and cell cycle changes in these 2D and 3D cultures. Seeding of A549 cells on two-dimensional tissue culture T-75 flasks (2D TC) was used as a reference standard. Other T-75 flasks were coated with collagen type I (2D Coll) as a control for collagen-based 3D models. Three-dimensional culturing has been performed in two different ways: one with a special instrument, the BioLevitator system, where cells were levitated and the other was collagen embedding in RAFT system. To generate different types of multicellular spheroids in the BioLevitator system, microcarrier free (3D Spheroid) and microcarrier based models were used (bead-based 3D Cytodex3 and 3D Nutrisphere, Figure 1). The cultivation of cells was performed under the same conditions: surface/volume ratio, cell culture media, glucose concentration, pH, pCO2 and pO2 tension either for 2D or 3D models. Representative images illustrate A549 cells in different cultures for 4 (Figure 1A) and 9 days (Figure 1B), respectively.

**Figure 2:**
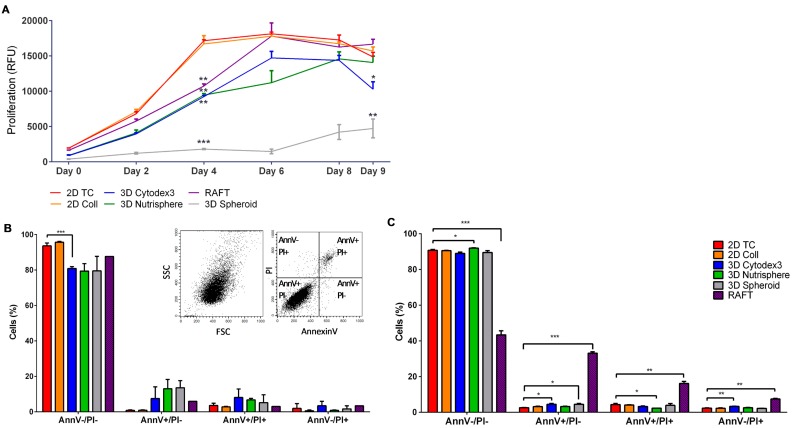
Proliferation rate, viability and apoptosis of A549 cells under different culture conditions. (**A**) Cells grow slowly in 3D culture than in 2D cultures on culture day 4 (3D spehorids *p* ≤ 0.0001; RAFT, 3D Cytodex3 and 3D Nutrisphere *p* ≤ 0.01), but on day 9 only the 3D Spheroid (*p* ≤ 0.01) and 3D Cytodex3 (*p* ≤ 0.05) showed significantly lower cell numbers than 2D cultures. (n = 3, paired t-test). The viability of cells (AnnV−/PI− living population) remained around 80–90% at day 4 (**B**) and day 9 (**C**), only RAFT culturing resulted in 50% decrease in viability at day 9. Insert shows representative dot plots of single cells for SSC-FSC (Side scatter-Forward scatter, B left insert) and for quadrants detecting living cells (AnnV−/PI−), early apoptotic cells (AnnV+/PI-), late apoptotic cells (AnnV+/PI+) and necrotic cells (AnnV−/PI+) (B right insert). Data are mean ± SD of three replicates.

Using resazurin cell proliferation assay the growth kinetics of differently formed 3D spheroids and 2D cultures were compared. The assay showed that cells in 2D cultures (2D TC, 2D Coll) reduced resazurin to resorufin more intensely proportionally with a higher number of cells than any of the used 3D models (Figure 2A). Both monolayer 2D cultures reached the plateau phase on day 4. The 3D spheroids grown on the surface of the Nutrisphere’s magnetic beads (3D Nutrisphere) reached the plateau only on day 8, while the spheroids grown on Cytodex3 microcarriers (3D Cytodex3) or the ones embedded into ECM matrix (RAFT) reached the same state on day 6. On the other hand, the carrier-free 3D Spheroids did not reach stationary phase in the duration of the experiment. These spheroids cultured without microcarriers grew very slowly and formed only small multicellular spheroids consisting of 50–100 cells. Thus, these spheroids were in an early stage of multicellular tumor formation (Figure 2A).

**Figure 3:**
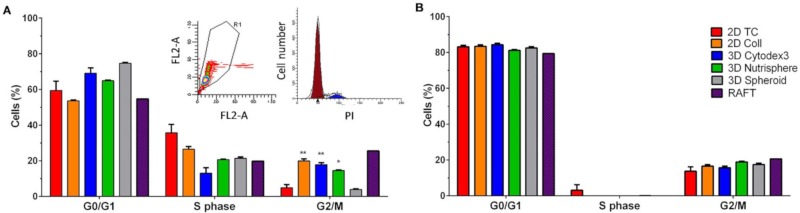
Cell cycle phase distribution of A549 cells cultured under different conditions at the 4^th^ (**A**) and the 9^th^ culturing days (**B**). Insert shows PI signal of single cells FL2-A (area)-FL2-W (width) gating out aggregates (**A** left insert) and a representative image of cell cycle phases (**A** right insert). Data are mean ± SD of three replicates except RAFT where 12 collagen discoids were pooled.

At two timepoints, when the growth of 2D cultures reached the plateau stage (day 4) and at the end of the experimental period (day 9) (Figure 2A), six types of 2D and 3D in vitro cultures were harvested as single cell suspension for flow cytometric analysis of viability (apoptosis) (Figure 2B,C) and cell cycle phase distribution (Figure 3). The viability of cells calculated from the percentage of cells without PI staining remained above 90% at both day 4 (Figure 2B) and day 9 (Figure 2C) except RAFT collagen embedding. Flow cytometric analysis showed that RAFT culturing resulted in 50% decrease in viability at day 9 (Figure 2C).

The ratio of G0/G1 and S (DNA synthesis) cell cycle phases did not differ significantly among the used 2D and 3D culture conditions either at day 4 (Figure 3A) and day 9 (Figure 3B). Cultures of 2D Coll, 3D Cytodex3, 3D Nutrisphere and RAFT showed higher population of cells in G2/M at day 4 (Figure 3A) which was not present at day 9 (Figure 3B).

### 3.2. Selection of Genes with Differential Expression In Vivo and in 3D Models Compared to Monolayer Cultures

Gene expression analysis was carried out in two steps. First gene expression changes were determined between in vitro monolayer culture and in vivo A549 xenograft cancer cells by high throughput TaqMan^®^ OpenArray^®^ Human Cancer Panel. Next, genes with differential expression were selected from the first analysis and were measured on the LightCycler 1536 HTS qPCR System for each in vitro 2D and 3D culture. Nanocapillary, quantitative real-time PCR (qRT-PCR) has been previously successfully used in our laboratory in toxicogenomics screening to cluster toxic compounds [31] and to predict organ-specific toxicity [35]. Here we performed the comparative investigation of gene expression of murine A549 xenografts (80–100 mm^3^) and monolayer cultures (A549 cell maintained in standard Petri-dish with 70% confluence) using a commercial Cancer Panel for 624 genes and 24 housekeeping genes (listed in Supplementary Materials File 1) in order to pre-select candidates for the subsequent qRT-PCR analysis on all 2D and 3D culture conditions under investigation. The TaqMan^®^ OpenArray^®^ Human Cancer Panel contains validated markers for pluripotency, DNA repair, angiogenesis, cell adhesion, apoptosis, ECM and cell cycle. Sixty genes which showed overexpression (29 genes, Table 2, pink) or downregulation (31 genes, Table 2, green) in A549 non-small cell lung tumors compared to A549 monolayer cultures have been selected for further studies. Expression data (ΔΔCt ± SD, *p*) of A549 xenograft compared to monolayer cultures for the whole panel (648 genes) are available in Supplementary Materials File 1.

**Figure 4:**
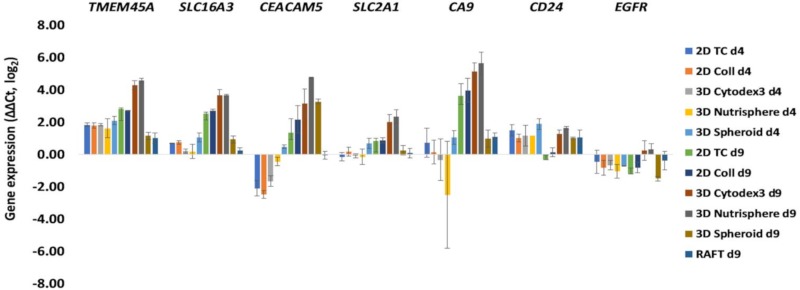
Gene expression changes after 4 (d4) and 9 days (d9) of culture under different 2D and 3D conditions. Selected genes having cell surface localized protein product and well characterized antibody available on the market: TMEM5A, SLC16A3 (MCT4), CEACAM5, SLC2A1 (GLUT1), and CA9 showed 16–32 times overexpression in 3D Cytodex3 and 3D Nutrisphere compared to 2D monolayer. Data are mean ± SD of three replicates.

Primer pairs were designed for the in vivo differentially expressed 60 genes identified by Open Array nanocapillary qRT-PCR, and *SLC2A1* (GLUT1) and *SLC16A3* (MCT4) in order to investigate the transcriptome of different 2D and 3D cultures by 1536 well high-throughput qRT-PCR (list of primer pairs can be found in Supplementary Materials File 2). Two key players of tumor cell metabolism, the lactate transporter *SLC16A3* (MCT4) and glucose transporter *SLC2A1* (GLUT1) were included because these probes were not present in the cancer panel, but it has been reported that high MCT4 and GLUT1 expression is associated with poor overall survival of adenocarcinoma patients [36]. Both early (day 4,) and late (day 9,) culture time points were analyzed (Figure 4). Gene expression data (ΔΔCt ± SD, *p*) of different 2D and 3D cultures can be found in the Supplementary Materials File 3. The characterization of genes in 2D and 3D cultures under investigation facilitated the design of an antibody panel for the subsequent single cell mass cytometry. Markers localized to the cell surface with well-characterized antibodies available on the market were selected. While *SLC16A3* (MCT4), *SLC2A1* (GLUT1) and *CA9* have been reported in metabolic reprogramming of malignant cells [37], the others have been shown to support tumor progression and chemoresistance via maintaining cancer stemness, epithelial-mesenchymal transition: *CEACAM5, CD24* [38,39] or via driving proliferation: *TMEM45A* [40], *EGFR* [41]. With the exception of *EGFR*, all markers showed 16–32-fold overexpression in three-dimensional bead-based cultures: the 3D Cytodex3 and 3D Nutrisphere compared to 2D monolayer in long-term cultures (d9) (Figure 4). At the day 9 collagen embedded RAFT and carrier-free spheroids behaved differently from bead-based cultures with very little change, except *CEACAM5,* where 3D Spheroids showed 8 times induction of expression (Figure 4). Gene expression analysis was not performed on RAFT d4 samples due to the limited amount of isolated RNA from cultured cells.

**Figure 5:**
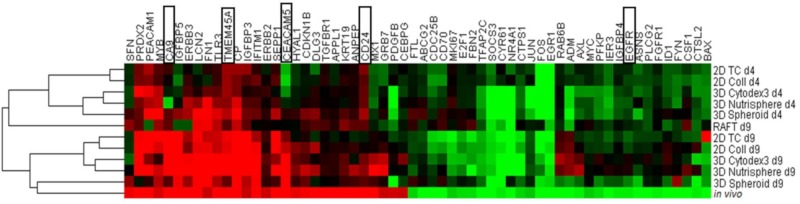
Long-term (day 9, d9) maintenance of 3D cultures (3D Spheroid, 3D Nutrisphere, 3D Cytodex3) mimic the in vivo situation better. The highlighted genes (black squares) were selected for subsequent analysis by single cell mass cytometry. Cluster analysis (hierarchical) was performed by Gene Cluster 3.0 software from expression data (ΔΔCt, log2 values) of presented samples.

In order to determine the closest culture method that mimicked the in vivo gene expression profile of non-small cell lung cancer cells, hierarchical clustering was performed on both days 4 and 9 of all the studied 2D and 3D culture methods (Figure 5). Short-term cultures (d4) of 2D TC, 2D Coll, 3D Cytodex3, 3D Nutrisphere, and 3D Spheroid represent a sub-cluster separate from long-term cultures and xenografts (*in vivo*) (Figure 5). Long-term (d9) maintenance of three-dimensional cultures (3D Spheroid, 3D Nutrisphere, 3D Cytodex3) clustered close to xenografts suggesting a closer relation to the in vivo situation (Figure 5). Cultures which are difficult to handle for high number of cells (RAFT) or difficult to standardize (3D Spheroids) were ignored in the subsequent experiments.

### 3.3. Single Cell-based Profiling Provides a Characteristic Map of Lung Cancer Markers

**Figure 6:**
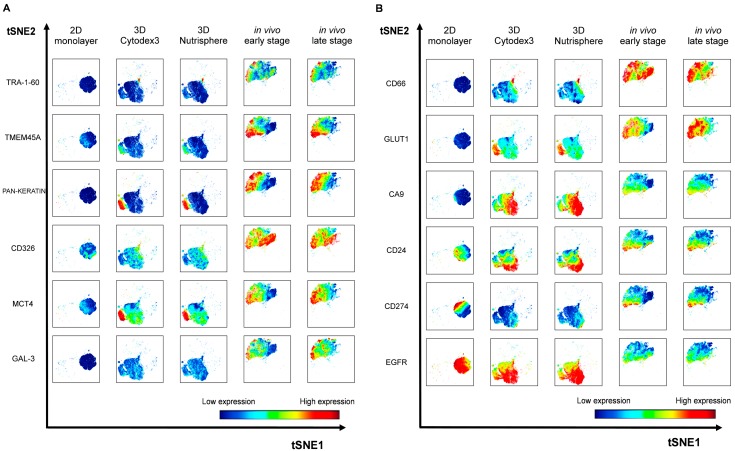
Representative multidimensional visualization of stochastic neighbor embedding (viSNE) analysis of 12 protein markers at single cell resolution in 2D, 3D (3D Cytodex3 or 3D Nutrisphere) cultures and in vivo (early or late stage) tumors. The analysis was performed within 4.5 × 10^4^ HLA-A,B,C positive cells in case of all conditions in order to identify human A549 cells in the xenografts and exclude murine stromal cells. (iterations = 1000, perplexity = 30, theta = 0.5).

Twelve cancer markers were studied by single cell mass cytometry from long-term (9 days) 2D monolayer and 3D Cytodex3, 3D Nutrisphere cultures and compared to early and late stage solid A549 tumors. Seven protein markers: TMEM45A, MCT4, CD66 (CEACAM5), GLUT1, CA9, CD24, EGFR were selected by their gene expression profile or their known relevance detailed above. Five additional proteins were studied: carcinoma stem cell markers: TRA-1-60 [42], CD326 (epithelial cell adhesion molecule, EpCAM) [43], galectin-3 (GAL-3) [44], the immune checkpoint inhibitor CD274 (programmed cell death ligand-1, PD-L1) [45] and carcinoma marker pan-keratin (cytokeratins) [46]. The relevance of the studied proteins was validated in two other NSCLC cell lines (H1975 and H1650) by mass cytometry (unpublished data). Although keratins are the building blocks of type I and II intermediate filaments of epithelial cells [46], it has also been published that these can localize to the outer surface of cancer cells to enhance cell adhesion to the ECM [47,48]. Representative multi-dimensional data analysis (visualization of stochastic neighbor embedding, viSNE, [34]) reveals cell-relatedness based on common marker expression by simultaneous analysis of all 12 markers at single cell resolution (Figure 6).

Single cells are mapped based on the expression of all 12 markers within each condition. Analysis was performed within 4.5 × 10^4^ HLA-A,B,C positive cells in case of all conditions in order to determine human A549 cells in the xenografts and exclude murine stromal cells. A549 cells (2D monolayer) were previously tested for HLA-A,B,C expression with 99.9% positivity (Supplementary Figure S4). The expression level of each marker is coded by the shown color-scale with blue representing low, while red, high values (Figure 6). Nine out of twelve markers (TRA-1-60, TMEM45A, pan-keratin, CD326, MCT4, GAL-3, CD66, GLUT1, CA9) were absent or showed relatively very weak expression in the 2D monolayer culture of A549 cells (Figure 6A,B, first columns). The pattern of three markers showed intra-cell line heterogeneity of standard Petri-dish based cultures by moderate (CD24, CD274) or strong (EGFR) protein load on the cell surface (Figure 6B, first column). Single cell proteome profiles of each three-dimensional culture (3D Cytodex3 and 3D Nutrisphere) represent a transition from 2D to the in vivo situation by intermediate marker expression in case of 9 proteins (TRA-1-60, TMEM45A, pan-keratin, CD326, MCT4, GAL-3, CD66, GLUT1, CD274) (Figure 6A,B, second and third columns). In bead carrier-based systems, three markers (CA9, CD24, EGFR) were exposed to the cell surface higher to in vivo (Figure 6B, second and third columns). All twelve markers drew the map of lung cancer cells in vivo as a different islet from the population of cells from 2D and 3D samples with a unique pattern reflecting intra-tumor heterogeneity on viSNE plots (Figure 6A,B, fifth columns).

**Figure 7:**
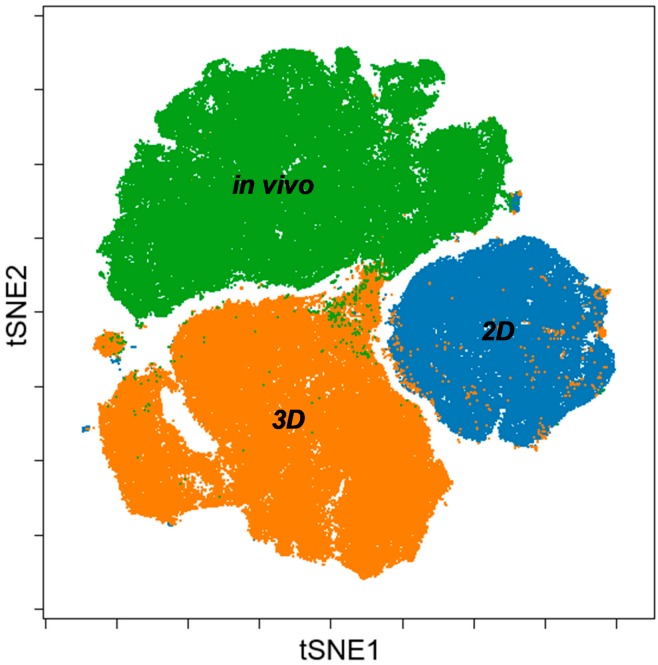
Merging viSNE graphs of multiparametric single cell mass cytometry data (12 parameters) of 2D, 3D and in vivo samples delineates a map with three different islands of 2D, 3D, and in vivo conditions.

Merging viSNE graphs of 2D, 3D and in vivo samples by multiparametric (12 proteins) single cell mass cytometry results delineate a map with three different ‘islands’ representing 2D, 3D and in vivo conditions with minimal overlap (Figure 7). Both segmentation and the area of the maps are proportional with heterogeneity of single cells in terms of the expression of the twelve studied tumor markers within a cohort. Standard 2D as the smallest tSNE island represents the poorest heterogeneity far from the 3D or in vivo condition.

**Figure 8:**
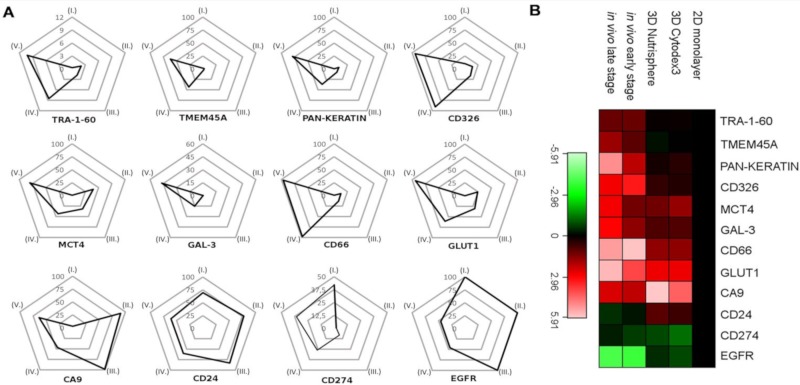
(**A**) Trajectories tend to localize to early and late stage tumors in the case of TRA-1-60, TMEM45A, pan-keratin, CD326, MCT4, GAL-3, CD66, and GLUT1. Percentage of HLA-A,B,C positive cells (A549) from a representative experiment were plotted on trajectories among five different conditions: (I) 2D monolayer, (II) 3D Cytodex3, (III) 3D Nutrisphere, (IV) early and (V) late stage in vivo adenocarcinoma. (**B**) Heatmap of mass cytometry data regarding protein density at single cell resolution among the five different conditions normalized to 2D monolayer (green: low, red: high expression).

In order to quantify marker expression at population level, percentage of HLA-A,B,C positive cells (A549) from a representative experiment were plotted on trajectories of radar plots among five different conditions: (I) 2D monolayer, (II) 3D Cytodex3, (III) 3D Nutrisphere, (IV) early and (V) late stage in vivo adenocarcinoma (Figure 8A). The trajectories show the percentage of A549 NSCLC (gated on HLA-A,B,C positive cells in order to exclude murine stroma) cells in the range of 0–100% for the positivity (expression) of each studied marker (Figure 8A). The following proteins determine trajectories within the pentagram to localize to advanced cancer (Figure 8): TRA-1-60 (9%), TMEM45A (37.5%), pan-keratin (50%), CD326 (100%), MCT4 (50%), GAL-3 (30%), CD66 (100%), GLUT1 (100%) (Figure 8A). Cells positive for CA9 have the highest population (100%) in 3D models probably due to the lack of circulation. Compared to monolayer condition, cancer stem cell marker CD24 and EGFR decreased in vivo from 70% to 35% and 100% to 25%, respectively. CD274 positive cells were 40 % in 2D and 25 % in vivo with weak expression in 3D models (Figure 8A).

Regarding protein density at single cell resolution, median values in all channels within HLA-A,B,C positive cells were normalized to reference 2D monolayer sample and visualized as a heatmap of studied proteins (Figure 8B). Three-dimensional cultures represent a transition from 2D to in vivo situation in terms of lung cancer marker expression, only TRA-1-60, TMEM45, pan-keratin and CD326 have intensity values (median) much below the value of the cells in vivo (Figure 8B).

## 4. Discussion

Genomics and proteomics opened new avenues for drug discovery with the emergence of novel therapeutic and diagnostic targets. Recent achievements in drug discovery with the combination of innovative 3D cell culture techniques yielded high-throughput screening (HTS) methodologies of 3D cellular assays in the pre-clinical phase of the drug discovery pipeline. These 3D HTS assays provide information not only on a general cellular response (cytotoxicity) to a given drug, but could map a signal transduction machinery or monitor the cellular response at transcriptional/translational level in a model system closer to the in vivo situation [49]. Monolayer cultures are oversimplified models of a multicellular organism. Beyond their benefits, they fail to present the in vivo situation in several aspects such as the flow of cellular metabolites, partial oxygen tension, the gradient of soluble mediators, whereas 3D tissue culture models have a closer resemblance to the in vivo conditions [50]. In 3D models oxygen and nutrient deprivation results in higher glycolytic activity, elevated autophagy and necrosis induced by anaerobic conditions [51,52,53,54]. Active signaling pathways are also different between 3D and 2D cultures for the lack of complexity of cell-cell and cell-ECM connections (integrins, proteoglycans) present in multicellular organism [55,56]. Different studies show that multicellular spheroid formation highly influenced extracellular matrix protein expression [57,58] and relevant gene expression levels as well [59,60].

In order to find the most suitable 3D culture method to mimic in vivo tumor biology we performed comparative analysis of different 2D (2D standard Petri-dish; tissue culture-treated T-75 flasks, 2D TC; type I collagen coated plates, 2D Coll); 3D bead-based (3D Cytodex3, 3D Nutrisphere); 3D carrier-free (3D Spheroid) and collagen embedding (RAFT) cell culture methods (Figure 1). These culture methods were compared to A549 xenograft tumors (in vivo). Both short-term (day 4) and long-term (day 9) cultures were analyzed with early stage and late stage adenocarcinoma.

The investigation of the proliferation rate in different culture methods revealed that carrier-free spheroids divided less frequently with the longest lag-phase, and viability was hampered in long-term RAFT cultures (Figure 2). These two 3D culture methods were excluded from our single cell experiments, due to their difficult handling and standardization of spheroid size. On the contrary, bead-based systems offer a constant and calculated surface/volume ratio. Both 3D Cytodex3 and Nutrisphere beads can be counted using standard methods, therefore the density of beads can be controlled for each experiment. Cell cycle phase distribution showed moderate changes in only short-term cultures (day 4), but not in long-term (day 9) with significant enrichment in the G_2_/M phase in 2D Coll, 3D Cytodex3 and 3D Nutrisphere cultures (Figure 3). Gene expression analysis by nanocapillary qRT-PCR (624 genes) and 1536 well high-throughput qRT-PCR (62 genes) resulted in the selection of lung cancer markers associated with higher (*TMEM45A, SLC16A3, CD66, SLC2A1, CA9, CD24*) or lower (*EGFR*) expression in vivo or in 3D models compared to monolayer cultures (Table 2, Figure 4 and Figure 5). Additionally, TRA-1-60, pan-keratins, CD326, Galectin-3, CD274 with known clinical significance were also included into the panel. Adenocarcinomas express keratins (K) such as: K8, K18, K19 and in some cases also K7 and K20 as building blocks of intermediate filaments [46]. Interestingly, these cytokeratins also localize to the cell surface of carcinoma cells to enhance adherence to ECM [47,48]. Therefore, an anti- pan-keratin antibody (clone C11) was used for cell surface labeling which recognizes keratins 4, 5, 6, 8, 10, 13 and 18 [61].

The implemented multidimensional single cell proteome profiling revealed that 3D (Cytodex3 and Nutrisphere) cultures represent a transition from 2D to in vivo situation by intermediate marker expression of TRA-1-60, TMEM45A, pan-keratin, CD326, MCT4, Gal-3, CD66, GLUT1, CD274. In 3D systems CA9, CD24, EGFR showed higher expression than in vivo (Figure 6). Our multi-parametric single cell mass cytometry results delineated a map with different regions that represented 2D, 3D and in vivo conditions with minimal overlap (Figure 7). Our single cell study was able to detect the rate of heterogeneity in 2D, 3D cultures and in solid-tumor (Figure 6 and Figure 7). As a result, the following proteins were associated with advanced cancer: TRA-1-60 (9%), TMEM45A (37.5%), pan-keratin (50%), CD326 (100%), MCT4 (50%), GAL-3 (30%), CD66 (100%), GLUT1 (100%) (Figure 8A).

The complexity of an organism is far from cell culture systems, but bead-based 3D cultures provide a better representation of the in vivo conditions affording a more effective methodology for different molecular biology studies, as well as for screening of compound libraries for novel anticancer drug candidates.

## Figures and Tables

**Table 1:** Antibodies used for mass cytometry.

Catalogue Number	Supplier	Target	Metal Tag
3144017B	Fluidigm	HLA-A,B,C	144_Nd
3141006B	Fluidigm	CD326 (EpCam)	141_Pr
3148012B	Fluidigm	TRA-1-60	148_Nd
3149018B	Fluidigm	CD66-a,c,e	149_Sm
3156026B	Fluidigm	CD274 (PD-L1)	156_Gd
3162027A	Fluidigm	Pan-Keratin	162_Dy
3166007B	Fluidigm	CD24	166_Er
3170009B	Fluidigm	EGFR	170_Er
3153026B	Fluidigm	Galectin-3 (Gal-3)	153_Eu
MAB2188-100	R&D Systems	CA9	158_Gd
MAB1418	R&D Systems	GLUT1	154_Sm
sc-376140	Santa Cruz Biotech.	MCT4	171_Yb
orb357227	Biorbyt	TMEM45A	169_TM

**Table 2:** Gene expression analysis by nanocapillary qRT-PCR of 624 genes (TaqMan^®^ OpenArray^®^ Human Cancer Panel, QuantStudio™ 12K Flex) of A549 xenografts (in vivo) compared to 2D monolayer (Petri-dish) cultures resulted in the selection of sixty genes: 29 upregulated (pink) and 31 downregulated (green) for further studies. Significance (*p*) was calculated by Student’s t-test as pairwise comparison of ΔCt values.

Gene Symbol	Assay ID	ΔΔCt (log2)	SD	Significance (*p*)	Gene Symbol	Assay ID	ΔΔCt (log2)	SD	Significance (*p*)
***CEACAM5***	Hs00944025_m1	**9.11**	1.77	**0.0220**	***JUN***	Hs00277190_s1	**−2.34**	1.97	0.2740
***APPL1***	Hs00179382_m1	**8.17**	0.12	**0.0001**	***MYC***	Hs99999003_m1	**−2.42**	1.96	0.2306
***LCN2***	Hs01008571_m1	**5.58**	0.68	**0.0082**	***MKI67***	Hs01032443_m1	**−2.43**	0.45	**0.0249**
***SEPP1***	Hs01032845_m1	**5.26**	1.10	**0.0293**	***FTL***	Hs00830226_gH	**−2.51**	1.21	0.1293
***PRDX2***	Hs03044902_g1	**4.71**	1.45	0.0590	***CTPS***	Hs00157163_m1	**−2.53**	1.41	0.1831
***TGFBR1***	Hs00610318_m1	**4.38**	0.02	**0.0001**	***E2F1***	Hs00153451_m1	**−2.63**	1.58	0.1555
***CP***	Hs00236810_m1	**4.36**	0.08	0.1173	***PFKP***	Hs00242993_m1	**−2.64**	1.70	0.1746
***ANPEP***	Hs00952642_m1	**3.79**	2.19	0.2148	***FBN2***	Hs00266592_m1	**−2.65**	0.17	0.2083
***DLG3***	Hs00221664_m1	**3.72**	0.22	**0.0213**	***CYR61***	Hs00155479_m1	**−2.79**	2.20	0.2390
***CA9***	Hs00154208_m1	**3.71**	1.42	0.0886	***CTSL2***	Hs00822401_m1	**−2.81**	1.03	0.0769
***CD24***	Hs00273561_s1	**3.66**	0.54	**0.0182**	***EGFR***	Hs01076078_m1	**−2.82**	0.38	**0.0345**
***IFITM1***	Hs00705137_s1	**3.60**	0.25	**0.0057**	***IGFBP4***	Hs00181767_m1	**−2.83**	1.73	0.1601
***PECAM1***	Hs00169777_m1	**3.54**	0.00	**0.0217**	***FGFR1***	Hs00241111_m1	**−2.87**	1.74	0.2655
***MX1***	Hs00895608_m1	**3.53**	0.92	0.0702	***AXL***	Hs01064444_m1	**−2.87**	1.81	0.1685
***TMEM45A***	Hs01046616_m1	**3.49**	1.42	0.2538	***ASNS***	Hs00370265_m1	**−2.98**	1.02	0.0890
***KRT19***	Hs00761767_s1	**3.38**	1.35	0.0793	***SOCS3***	Hs02330328_s1	**−3.04**	1.01	0.1962
***TLR3***	Hs00152933_m1	**3.37**	0.48	**0.0330**	***IER3***	Hs00174674_m1	**−3.18**	1.78	0.2419
***ERBB3***	Hs00176538_m1	**3.27**	0.95	0.0524	***CDC25B***	Hs01550934_m1	**−3.20**	1.51	0.1116
***IGFBP5***	Hs01052296_m1	**3.18**	1.03	0.0563	***BAX***	Hs00180269_m1	**−3.32**	1.22	0.0804
***CDKN1B***	Hs00153277_m1	**2.91**	0.22	**0.0217**	***TFAP2C***	Hs00231476_m1	**−3.60**	1.58	0.0921
***SFN***	Hs00968567_s1	**2.83**	0.61	**0.0329**	***ABCG2***	Hs01053790_m1	**−3.66**	0.85	**0.0368**
***HYAL1***	Hs00201046_m1	**2.74**	0.38	**0.0096**	***NR4A1***	Hs00374226_m1	**−3.66**	0.83	**0.0250**
***MYB***	Hs00920554_m1	**2.54**	1.61	0.2887	***RAB6B***	Hs00981572_m1	**−3.66**	0.89	**0.0446**
***PDGFB***	Hs00966522_m1	**2.47**	0.47	0.0853	***CD70***	Hs00174297_m1	**−3.94**	0.00	0.1385
***ERBB2***	Hs01001580_m1	**2.40**	1.25	0.1180	***CSF1***	Hs00174164_m1	**−4.03**	0.19	**0.0107**
***GRB7***	Hs00918009_g1	**2.39**	0.67	0.3032	***FYN***	Hs00176628_m1	**−4.09**	1.86	0.0988
***FN1***	Hs01549976_m1	**2.38**	1.25	0.1188	***PLCG2***	Hs00182192_m1	**−4.13**	2.19	0.1465
***CEBPG***	Hs00156454_m1	**2.37**	1.99	0.5677	***EGR1***	Hs00152928_m1	**−4.19**	2.60	0.1582
***IGFBP3***	Hs00181211_m1	**2.34**	1.03	0.1114	***ID1***	Hs03676575_s1	**−4.38**	0.22	**0.0155**
					***ADM***	Hs00181605_m1	**−4.51**	0.10	**0.0013**
					***FOS***	Hs00170630_m1	**−4.61**	2.40	0.1254

## Supplementary Materials

The following are available online at https://www.mdpi.com/2073-4409/8/9/1093/s1, File S1: Cancer Panel Assay List; File S2: qRT-PCR (1536) primer list; File S3: qRT-PCR data, Figure S4: HLA-A,B,C+ population of A549 cells.
