# Supplementary figures and images for: Single Cell Mass Cytometry of Non-Small Cell Lung Cancer Cells Reveals Complexity of In Vivo and Three-Dimensional Models over the Petri-Dish
Source: Cells. 2019 Sep 16;8(9):1093. doi: 10.3390/cells8091093 (PMC6770097; doi:10.3390/cells8091093)

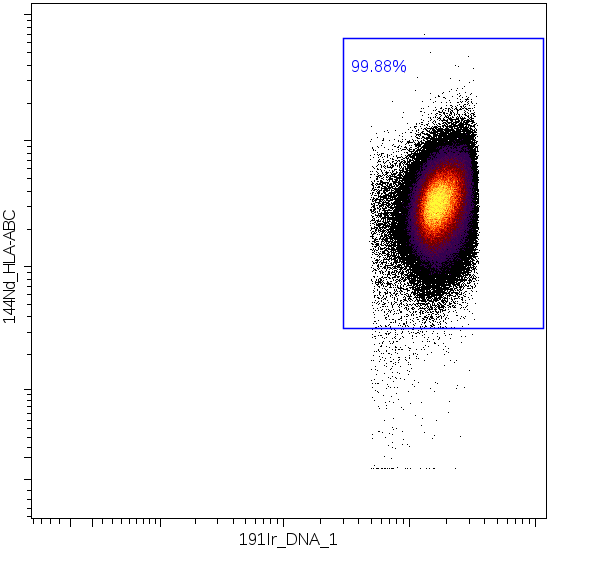

Supplement: Supplementary file 1 [file cells-08-01093-s001.zip › Supplementary/Supplement 4.JPG]
